# *Candida auris* Outbreaks: Current Status and Future Perspectives

**DOI:** 10.3390/microorganisms12050927

**Published:** 2024-05-01

**Authors:** Silvia De Gaetano, Angelina Midiri, Giuseppe Mancuso, Maria Giovanna Avola, Carmelo Biondo

**Affiliations:** Mycology Laboratory, Department of Human Pathology, University of Messina, 98125 Messina, Italy; sdegaetano6@gmail.com (S.D.G.); amidiri@unime.it (A.M.); mancusog@unime.it (G.M.); maria.avola@studenti.unime.it (M.G.A.)

**Keywords:** antifungal resistance, infection control, nosocomial spread, screening, identification

## Abstract

*Candida auris* has been identified by the World Health Organization (WHO) as a critical priority pathogen on its latest list of fungi. *C. auris* infections are reported in the bloodstream and less commonly in the cerebrospinal fluid and abdomen, with mortality rates that range between 30% and 72%. However, no large-scale epidemiology studies have been reported until now. The diagnosis of *C. auris* infections can be challenging, particularly when employing conventional techniques. This can impede the early detection of outbreaks and the implementation of appropriate control measures. The yeast can easily spread between patients and in healthcare settings through contaminated environments or equipment, where it can survive for extended periods. Therefore, it would be desirable to screen patients for *C. auris* colonisation. This would allow facilities to identify patients with the disease and take appropriate prevention and control measures. It is frequently unsusceptible to drugs, with varying patterns of resistance observed among clades and geographical regions. This review provides updates on *C. auris*, including epidemiology, clinical characteristics, genomic analysis, evolution, colonisation, infection, identification, resistance profiles, therapeutic options, prevention, and control.

## 1. Introduction

The incidence of human fungal infections is on the rise, affecting billions of people worldwide [1]. Annually, these infections cause the deaths of over 1.5 million people [2]. On 25 October 2022, the World Health Organization (WHO) published a list of 19 priority fungal pathogens that pose the greatest threat to public health [3,4]. The WHO has classified the priority fungal pathogens into three categories: critical, high, and medium priority [2,5]. *Candida* spp. are the primary cause of invasive fungal infections (IFIs) among fungal pathogens [6,7,8,9]. The increased use of fluconazole and other antifungals as prophylaxis or therapy for IFIs has increased the incidence of non-albicans *Candida* species-invasive infections [10]. Multidrug-resistant *Candida* spp that are emerging include *C. glabrata*, *C. krusei*, *C. lusitaniae*, members of the *C. guilliermondii complex*, *C. kefyr*, members of the *C. haemulonii complex*, and *C. auris* [11]. The latter, a multidrug-resistant fungal pathogen, is classified as a critical priority by the WHO [12]. This formal recognition by the WHO is intended to provide guidance for research, development, and public health action in the context of invasive fungal diseases. In light of the growing prevalence of multidrug-resistant *C. auris* strains, it is imperative to develop novel therapeutic strategies that encompass the development of new antifungal agents and the combination of existing drugs. *C. auris* is a member of the ascomycetous (hemiascomycetes) *Clavispora* clade in the *Metschnikowiaceae* family of the order *Saccharomycetales*. The term ‘auris’ is derived from the Latin word for ‘ear’. It was first discovered in the ear canal of a 70-year-old Japanese patient who was hospitalised in 2009, although the first retrospective isolation dates back to 1996 [13,14]. Identification was performed by analysing the sequence of the D1/D2 domain of the 26S rDNA gene and the ITS region using phylogenetic analysis. This allowed clinicians to distinguish *C. auris* from its closest phylogenetically related species, *C. heamulonii*, for the first time [15,16]. The first recorded case of *C. auris* in Europe dates back to 2007 and belongs to the South Indian clade (clade I) [17]. Out of 15,271 *Candida* isolates collected between 2004 and 2015, only 4 were identified as *C. auris*. All four were collected after 2009 [18,19].

Articles published between 2019 and 2023 were searched for recent updates on *C. auris*. The search was limited to publications in the English language and included Medline, Scopus, and Global Health. The search term ‘*C. auris*’ was used. This review objectively and clearly discusses the most recent updates in *C. auris*, including epidemiology, clinical characteristics, genomic analysis, evolution, colonisation, infection, identification, resistance profiles, therapeutic options, prevention, and control. 

### 1.1. Epidemiology

Shortly after it was first described in 2009, *Candida auris* isolates were reported to cause not only otitis media, as the name suggests, but invasive infections leading to hospital outbreaks. In 2018, the European Centre for Disease Prevention and Control (ECDC) updated its rapid risk assessment for *C. auris* in healthcare settings due to a significant increase in the number of cases detected in European countries (Table 1). 

The number of reported cases in the US increased from 476 in 2019 to 1471 in 2021. Similarly, in Europe, the reported cases rose from 335 to 655 in the 2020/2021 biennium [20]. Following an outbreak in northern Italy, a further risk assessment was carried out in 2022 [21]. *C. auris* has been detected in almost 100 hospitals in South Africa, causing large outbreaks and accounting for approximately 10% of candidemia cases [22]. In India, in 27 intensive care units (ICUs), *C. auris* has been implicated in 5% of cases of candidemia [23,24]. The diagnosis of *C. auris* infection can be challenging due to the lack of specific symptoms that are exclusively characteristic of this fungal pathogen. Furthermore, *C. auris* should be included in the list of reportable pathogens associated with healthcare-associated infections, as colonised patients may act as a potential source of transmission of *C. auris* to other patients within healthcare facilities and may also be at risk of invasive infections. The prevalence of *C. auris* in the community is currently unknown and there is no routine screening for this fungal pathogen upon admission to hospital [25]. Two separate studies conducted in the UK and the USA found low rates of carriage upon admission, which were only identified in those who had previous exposure to a hospitalised environment [20,26,27]. 

### 1.2. Clinical Characteristics

**Figure 1:**
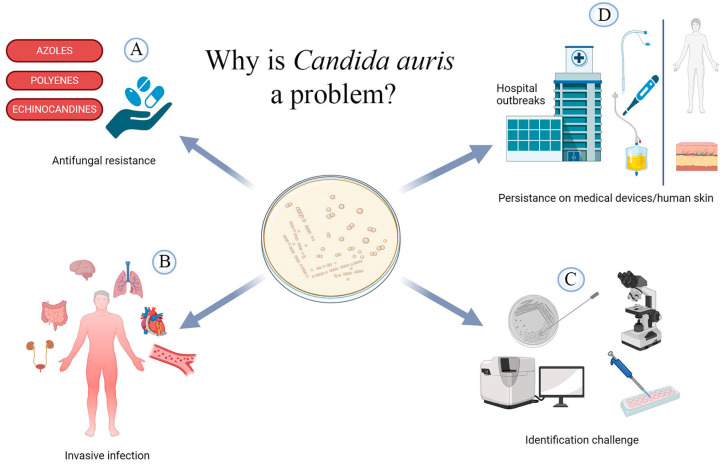
The emergence of *Candida auris* as a human pathogen is a significant concern for several reasons. (**A**) Most *C. auris* isolates are resistant to antifungals commonly used against other *Candida* species, and multidrug-resistant strains have recently emerged. (**B**) *C. auris* is capable of causing severe invasive infections, such as bloodstream infections, meningitis, endocarditis, intra-abdominal infections, respiratory infections, and urinary tract infections [48]. (**C**) Identifying *C. auris* isolates can be challenging due to the limitations of commonly used diagnostic tools, including cultural and biochemical methods. This can lead to improper therapies, multidrug resistance, and fatal outcomes. (**D**) *Candida auris* is capable of persisting on medical devices, such as catheters, thermometers, endotracheal tubes, and human skin, facilitating its transmission among patients and causing hospital outbreaks. This is true even in the presence of commonly used disinfectants.

This microorganism causes severe invasive infections with high mortality rates worldwide, particularly among hospitalised and immunocompromised patients [28,29,30,31,32]. Despite the absence of global data regarding the incidence of *Candida auris* and mortality rates, the notable rise in clinical cases is an emerging concern due to the organism’s distinctive characteristics (Figure 1) [33]. Firstly, it displays an unparalleled level of resistance to multiple antifungal agents commonly used to treat other *Candida* infections, which severely limits therapeutic options [34]. Recently, pan-antifungal resistant strains have also been reported [35]. Although the overall number of pan-resistant cases reported so far is low, their emergence is worrying [35]. Detection in axillary and groin swabs may indicate carriage rather than infection with a risk of transmission to others and later invasive infection [36,37,38]. Unlike other *Candida* infections, which are usually the result of autoinfection by the host flora, *C. auris* does not colonise mucosal surfaces or the gastrointestinal tract [39]. Instead, it has a distinct affinity for the skin, where it can persist for extended periods [39]. *C. auris* infections are frequently reported in the bloodstream and have also been linked to wound, catheter tip, and intra-abdominal infections (Figure 1) [40]. It has also been isolated from ear and respiratory samples, urine, bile, and jejunal biopsies [41]. Although the disease burden is lower in the paediatric population than in adults, there have been 256 reported cases in children, mostly from South Africa and South Asia [42]. Mortality rates for paediatric patients with *C. auris* bloodstream infections were up to 40%, which is lower than that of adult patients [42,43,44]. Healthcare-acquired transmission of *C. auris* mainly affects individuals with chronic illnesses, a history of other resistant pathogens, and invasive medical devices such as mechanical ventilation, tracheostomies, feeding tubes, and urinary catheters [30,45]. The halotolerance of *C. auris* enables it to survive on the skin, particularly in areas that are frequently exposed to high salinity and temperatures during periods of strenuous physical activity, such as the axilla and groin [46]. Effective disinfection and sterilisation are crucial in reducing the risk of contamination of reusable medical devices, which has been linked to significant mortality. The importance of these measures is highlighted by concerns about the transmissibility and persistence of infections [30,47].

### 1.3. Genomic Analysis 

**Figure 2:**
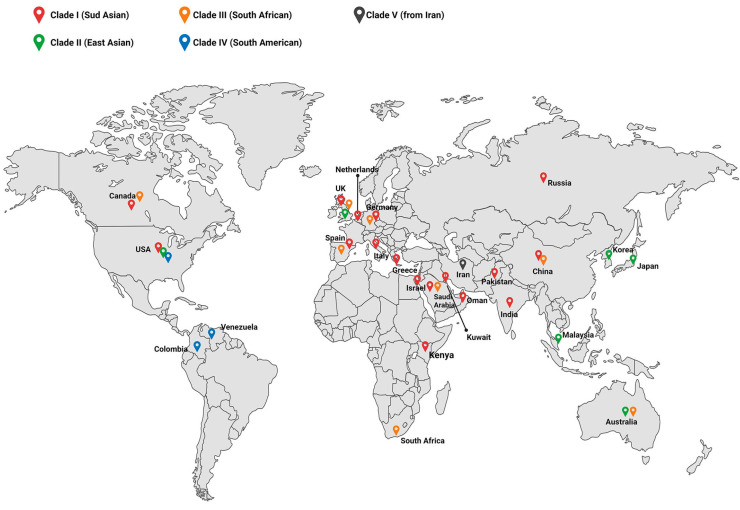
A map illustrating the global distribution of major clades of *C. auris*. Clade I is the most prevalent, with occurrences reported in 17 countries. These include India, Kuwait, the United Kingdom, the USA, Canada, Oman, Pakistan, Spain, China, Kenya, Russia, Israel, Germany, the Netherlands, Greece, and Italy. Clade II was identified in the USA, the United Kingdom, Japan, Korea, Malaysia, and Australia. Clade III was reported in eight countries: South Africa, the United Kingdom, China, Germany, Saudi Arabia, Spain, Australia, and Canada. Clade IV was identified in the United States, Colombia, and Venezuela. In contrast, clade V is the only clade reported exclusively in Iran.

WGS analyses indicate that *C. auris* emerged at the same time on several continents, with six distinct clades in South Asia, East Asia, Africa, and South America [49]. Clade I, the South Asian clade, was first discovered in India and Pakistan; clade II, the East Asian clade, was first discovered in Japan; clade III, the South African clade, was first discovered in South Africa; clade IV, the South American clade, was first discovered in Venezuela; and clade V was recently discovered in Iran [50]. *Candida auris* isolates frequently exhibit drug resistance, with patterns varying among clades and geographical regions [51]. Recently, two additional genetically distinct clades, both fluconazole-resistant, have been isolated in Iran [52]. Thousands of single nucleotide polymorphisms (SNPs) separate each clade, but strains within each clade are highly clonal, with an average of fewer than 70 SNPs separating two isolates within a clade [53]. Together with different geographical resistance mechanisms, it is likely that clones are expanding and evolving independently. Increasing antifungal selection pressure in humans, animals, and the environment may be driving the emergence of the pathogen. In a retrospective analysis conducted worldwide from 2009 to 2020, the most prevalent strain (clade I) was found to be South Asian and was identified in 17 countries [54]. The South African strain (clade III) followed, identified in eight countries, and the East Asian strain (clade II) was reported in only five countries. The South American strain (clade IV) was identified in three countries [54] (Figure 2).

### 1.4. C. auris and COVID-19

Previous research has indicated that patients with severe COVID-19 infection during the early stages of the pandemic were at an elevated risk of developing bacterial and fungal infections [32,55]. The COVID-19 pandemic may have contributed to the increased spread of *C. auris*. This could be due to the pressure on the global healthcare system and compromised infection prevention and control practices. Additionally, the pandemic may have hindered the detection of additional cases [56]. However, outbreaks of *C. auris* among severely ill COVID-19 patients during the peak of the pandemic were only observed in certain countries, such as Italy and the USA [21]. An epidemiological analysis conducted during the pandemic in the United Arab Emirates found an increase in COVID-19 and *C. auris* co-infection in hospitals with overwhelmed intensive care units [57]. Patients had significant underlying comorbidities and were treated with broad-spectrum antibiotics and immunosuppressive therapies. They also had indwelling urinary catheters and intravenous catheters [58]. An outbreak of *C. auris* was identified in an ICU with COVID-19 patients in India. The mortality rate was high. The time of infection onset ranged from 3 to 8 weeks after admission. Most patients had central venous and urinary catheters, required mechanical ventilation, and had underlying chronic diseases such as diabetes mellitus and hypertension [59]. During the pandemic, Spain and Italy experienced a significant increase in *C. auris* and multidrug-resistant organism infections, with some cases of nosocomial outbreaks in patients admitted to intensive care units with COVID-19 infection [60,61].

### 1.5. Insights into the Evolution of C. auris

**Figure 3:**
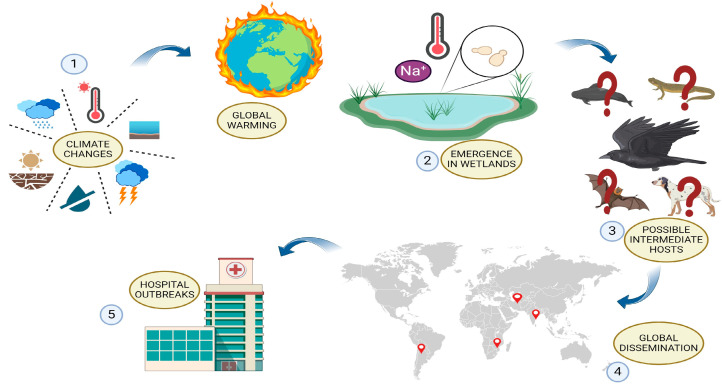
Transmission and evolutionary dynamics of *Candida auris*. (**1**) The impact of anthropogenic climate change is increasingly affecting infectious diseases worldwide. This is due to higher temperatures, sea level rise, droughts, and desertification. (**2**) It is possible that global warming acted as a selective pressure on non-pathogenic *C. auris* ancestors, favouring strains adapted to live in high-salinity regions and higher temperatures, such as wetlands. (**3**) The independent evolution of four geographically distinct *C. auris* clades may have been caused by the transportation of the fungus from its original environment to other areas of the planet by migratory birds or other intermediate hosts. (**4**) These clades have since spread globally. (**5**) It is possible that the fungus first colonised humans in rural areas before subsequently emerging in hospitals.

A puzzling feature of *Candida auris* is its rapid and simultaneous emergence on all continents. Several hypotheses have been proposed to explain its emergence. One of these is the use of an improved diagnostic tool to identify it. To test whether it had been misidentified before its recent emergence in 2009, the SENTRY Antifungal Surveillance Program was reviewed. The program includes 20,788 candidemia isolates gathered between 1997 and 2016 from Asia, Europe, Latin America, and North America [62]. Only a small number of presumptive *C. haemuloni* isolates collected prior to 2009 have been retrospectively re-identified as *C. auris* using MALDI-TOF mass spectrometry. These data confirm that the global spread of *C. auris* is a recent occurrence, with few reported cases prior to its initial report. Due to the significant increase in reports of *C. auris* across various regions worldwide, it was necessary to investigate whether it originated from a single location and then spread globally, possibly through human migration, or whether it originated independently in different countries. Whole genome sequencing (WGS) and single nucleotide polymorphism analysis, combined with epidemiological observations, have shown that genetically unrelated clonal populations of *C. auris* emerged independently and simultaneously in different geographical areas [63]. Genetically distinct clades of *C. auris* display high inter-clade genetic diversity, differing from each other by tens to hundreds of thousands of SNPs. However, their intra-clade diversity is much lower, ranging from tens to hundreds of SNPs, supporting the hypothesis of the independent emergence of *C. auris* in different parts of the world [64]. The different clades displayed unique clinical and microbiological characteristics. Nosocomial outbreaks and invasive infections were linked to clades I, III, and IV. This is consistent with the results of Bayesian molecular clock dating analyses, which estimated that the outbreak-causing isolates belonging to these three clades originated 36 to 38 years ago [65]. In contrast, clade II and clade V have primarily been associated with ear colonisation or infection [51]. However, the process through which this organism became a human pathogen on three continents remains unclear. Furthermore, the explanation for the recent and progressive acquisition of virulence factors is unconvincing as it does not account for their simultaneous emergence in different regions. The virulence capacity of a species depends on a set of pathogenicity determinants that should have occurred globally at the same time [64,66,67]. During the 20th century, human activity caused significant changes to the natural environment, resulting in climate change. As a consequence, the ecology of infectious diseases has been affected [68]. It has been hypothesised that the emergence of *C. auris* may be linked to a warmer climate (Figure 3). This theory suggests that higher ambient temperatures could have contributed to the proliferation of *C. auris* in the environment due to its higher thermal tolerance compared to other *Candida* spp. [69]. Thus, according to this theory, the thermotolerance and pronounced halotolerance of *C. auris* may have originated from a non-pathogenic environmental ancestor found in high-salinity regions, which through evolutionary adaptation was able to overcome the mammalian thermal barrier and cause infection [70]. *C. auris* has been found in various marine habitats, including public swimming pools, confirming its adaptability to different aquatic environments [70]. However, this does not explain why genetically distinct clades co-evolved in different regions worldwide. It has been suggested that birds may have contributed to the global spread of *C. auris* progenitors, enabling the independent evolution of different clades across continents (Figure 3). The recent discovery of *C. auris* in the oral cavity of a dog supports the hypothesis of zoonotic transmission [71]. The validation of the theory necessitates multidisciplinary research to clarify transmission dynamics and investigate potential environmental and animal reservoirs. It is important to consider the fungus’ morphogenetic flexibility, which enables it to adapt to various environments and persist.

### 1.6. The Dynamics of C. auris Colonisation and Infection

The risk factors for *C. auris* infection are not different from those of other *Candida* species [72]. These include prolonged stays in high-risk healthcare settings such as ICUs and severe underlying diseases such as HIV, neutropenia, chronic kidney disease, and diabetes mellitus [73]. Other important factors include the presence of catheters, mechanical ventilation, long treatment with antibiotics or antifungals, and invasive surgery [74]. 

*C. auris* is an opportunistic pathogen with a clinical spectrum similar to that of other Candida spp. [47,75]. The range of *Candida* infection varies from asymptomatic colonisation to superficial and invasive infection. The latter is most commonly associated with healthcare settings. *Candida auris* is not a commensal yeast, but it can persist on human skin and abiotic surfaces for extended periods [30]. This persistence has resulted in significant outbreaks in healthcare facilities, as the yeast is easily transmitted through contact with contaminated objects or through skin-to-skin contact with infected individuals [10]. According to the CDC, patients should undergo screening for *C. auris* colonisation if they have been admitted to a healthcare facility within the last year or if they have been admitted to a healthcare facility abroad and are infected with carbapenemase-producing Gram-negative bacteria [28]. This is because the co-colonisation of *C. auris* with these microorganisms is very common, particularly in countries with documented cases of *C. auris*. Although screening upon admission to hospital is not routine, there is considerable evidence to support the importance of screening all critically ill patients. This is particularly important for critically ill patients, such as those with chronic respiratory disease, who are at significant risk of colonisation, particularly in areas where *C. auris* is endemic [30,76]. Different studies have demonstrated that patients who are discharged to the community typically take around eight months to become colonisation-negative. However, patients who remain in healthcare facilities often continue to experience persistent colonisation [77,78,79].

### 1.7. Identification and Typing

**Figure 4:**
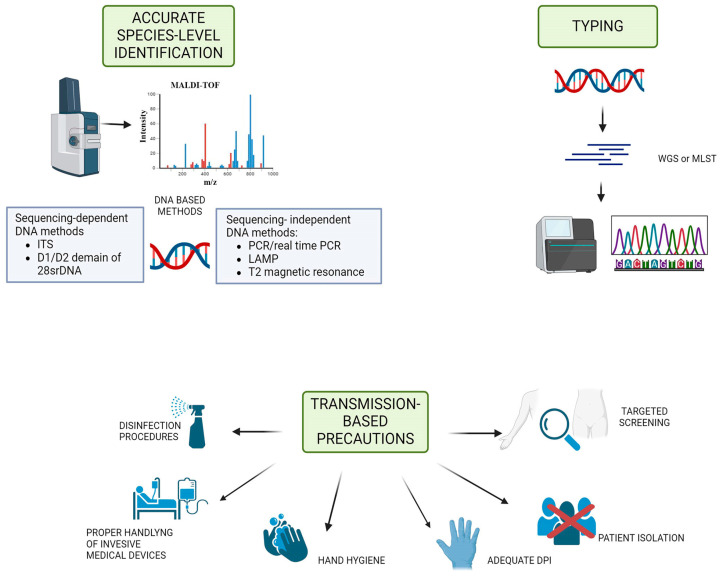
Schematic representation of prevention and control strategies for *C. auris* infections. Either MALDI-TOF MS or DNA-based methods, both sequencing-dependent and -independent, are recommended for species identification. For the control of hospital outbreaks, typing is necessary, requiring sophisticated diagnostic tools such as multilocus sequence typing (MLST) or whole genome sequencing (WGS). Once *C. auris* has been identified, transmission precautions should be put in place immediately.

The identification of *C. auris* by biochemical methods can be difficult and unreliable if the test has not been updated to include *C. auris*, due to phenotypic similarities with other species such as *C. haemulonii*, *C. duobushaemulonii*, *C. lusitaniae*, and *C. famata* [80]. Although commercial biochemical tests are commonly used to identify *C. auris*, they are not always effective in identifying all isolates. For example, the VITEX 2 XL (bioMérieux version 8.01) can detect *C. auris* isolates from the South American clade, but has limited ability to correctly identify *C. auris* from the African or East Asian clades [81]. An excellent alternative to standard fungal media for screening patients potentially colonised/infected with *C. auris* is CHROMagarTM Candida Plus. At 36 h of incubation, it has a sensitivity and specificity of 100%, with *C. auris* colonies appearing as a light blue colour with a blue halo [82]. Options for species identification include matrix-assisted laser desorption/ionisation time-of-flight (MALDI-TOF) mass spectrometry and molecular methods (Figure 4) [80]. The sequencing of different DNA loci in specific regions of ribosomal genes, such as 18S rDNA, 28S rDNA, or internal transcribed spacers ITS1 and ITS2, can be used for molecular identification of *C. auris* [83]. Real-time polymerase chain reaction (PCR) and loop-mediated isothermal amplification (LAMP) are alternative methods that can also be utilised [84]. However, in the investigation of an outbreak, higher-resolution methods such as whole genome sequencing analysis and typing by amplified fragment length polymorphism (AFLP) are needed to identify the route of disease transmission within a population and to provide information on the likely source [85]. Recent reports suggest that strains from more than one clade are found in most continents and countries, indicating that clades are more widely distributed. Therefore, the isolation area is no longer a reliable parameter for clade typing [86]. Investigations into potential outbreaks should begin as soon as a single case is detected in a hospital setting. Typing is essential in order to characterise the introduction and spread of the outbreak (Figure 4). Identifying the clade level will provide information on the strain’s antimicrobial resistance, virulence, biofilm production, and mortality rate [86]. This information is crucial as isolates from different geographical clades may differ in these properties. The identification of new and genetically distinct strains in *C. auris* is essential, and typing plays a significant role in this process. The presence of both MTLa and MTLα coupling types in each clade of *C.auris* raises concerns about the possibility of recombination between strains from different clades. Isolates from clades 1 and 4 exhibit the MTLa coupling type, while isolates from clades 2 and 3 exhibit the alternative MTLα coupling type [65,86]. Due to the mixed distribution of strains with different types of coupling, it is possible that genetically distinct hybrid profiles may emerge, which could be highly resistant or highly virulent.

### 1.8. Antifungal Drug Resistance in C. auris

Determining the most effective treatment for *C. auris* is difficult due to the absence of established susceptibility breakpoints [85]. Although clinical cut-off values for *C. auris* antifungal susceptibility tests have not yet been reported, it is known that high MICs for some antifungal agents, such as azoles, indicate that these compounds are not a viable therapeutic option for most clinical cases [34]. However, tentative CDC MIC breakpoints have been used for the following antifungal agents: fluconazole (≥32 µg/mL), voriconazole (≥2 µg/mL), posaconazole (≥2 µg/mL), itraconazole (≥2 µg/mL), anidulafungin (≥4 µg/mL), micafungin (≥4 µg/mL), caspofungin (≥4 µg/mL), and amphotericin B (≥2 µg/mL) [87]. According to these breakpoints, around 90% of *C. auris* isolates in the US are resistant to azoles, 30% are resistant to amphotericin B, and 3–5% are resistant to echinocandins. It is important to note that these percentages may vary and exhibit varying levels of resistance depending on the specific clade. Clade II exhibits the highest sensitivity to fluconazole, whereas isolates of clade I from the United Kingdom, India, and Pakistan demonstrate the highest resistance levels to fluconazole (97%) and amphotericin B (40–50%) [88]. Clades IV and III demonstrate the highest levels of resistance to echinocandins, reaching up to 7%. Echinocandins are crucial for treating *C. auris* infections and are the recommended first-line therapy for the most invasive *Candida* infections. Echinocandin resistance, particularly when combined with resistance to azole and amphotericin B (pan-resistance), is a significant clinical and public health concern [42,89]. Antifungal drug resistance in *C. auris* is acquired through various mechanisms, including the alteration of drug targets, increased efflux pump activity, or the activation of cellular stress response pathways [90]. These mechanisms are not unique to *C. auris* [91]. Fluconazole is a triazole derivative that interferes with the enzyme lanosterol α1-4 demethylase. This enzyme is encoded by the gene *ERG11* and is involved in ergosterol biosynthesis. Mutations in *ERG11*, such as *F126L*, *Y132F*, and *K143L*, are responsible for azole resistance. The K143R mutation is related to cross-resistance to azoles and could partially explain the high resistance to azoles exhibited by *C. auris* [92,93]. Furthermore, these mutations often coexist with the overexpression of genes encoding major facilitator efflux pumps (MDR) and ATP-binding cassette transporters for CDR, or their transcription factors (MDR1 and TAC1). Additionally, mutations in the *Upc2* gene lead to the overexpression of *ERG11* [94]. These mechanisms can act individually, sequentially, or simultaneously, resulting in the emergence of isolates with increasingly higher levels of azole resistance. Amphotericin B is a polyene that binds to ergosterol and creates pores at the membrane level. Resistance to this drug is primarily caused by modifications in the sterol composition of the membrane [95]. The mechanism of *C. auris* has not been extensively studied, but it is believed to be associated with modifications in the genes ERG2, ERG3, or ERG6, similar to other *Candida* species. These modifications result in a decrease in ergosterol content and the replacement of biosynthetic precursors, such as lichesterol, lansosterol, and fecosterol, which have reduced affinity for amphotericin B [96]. Echinocandins are lipopeptidic antifungal drugs that inhibit the enzyme β-(1-3)D-glucan synthase, which is encoded by the *FKS* genes (*FKS1, FKS2, FKS3*). In *C. auris*, resistance to echinocandins is achieved through point mutations in the *FKS1* gene (S639F, S639P, S639Y, F635P, and S635P). The S639 mutation is believed to provide pan-echinocandin resistance [64]. 

A molecular mechanism of resistance has been reported in a *C. auris* strain that is resistant to 5-fluorocytosine. This mechanism involves an SNP in the *FUR1* gene that leads to a change in the F211I residue in the enzyme uracil phosphoribosyl transferase. This enzyme is crucial for converting the drug into its active form [97]. Therefore, the development of new antifungal drugs that target these mechanisms in a broad range of fungal pathogens is crucial. It is estimated that approximately 50% of *C. auris* isolates from five continents are resistant to one or more classes of antifungal agents [38]. Although resistance to echinocandins has been rare, concerns have been raised due to evidence of transfer between healthcare facilities. Patients colonised with multidrug-resistant *C. auris* who are discharged into the community may subsequently receive treatment in another healthcare setting, leading to nosocomial transmission of *C. auris* [77,98]. The emergence of echinocandin-resistant isolates after prolonged exposure to anidulafungin or caspofungin during an outbreak is a cause for concern [99].

## 2. Therapeutic Options

The treatment of *Candida* infections varies depending on the site of the infection, the immune status of the patient, and the species of *Candida* responsible for the infection [100,101]. Current guidelines recommend echinocandin monotherapy as the form of empirical treatment prior to the results of susceptibility testing for candidemia and invasive candidiasis [102]. Caspofungin and micafungin are recommended for the treatment of adults and children over six months of age, while anidulafungin is only recommended for adults. This treatment is also recommended for *C. auris* isolates, which are commonly resistant to fluconazole [102]. Late complications may occur with echinocandin therapy and echinocandin-resistant strains may emerge during *C. auris* treatment [103]. Therefore, it is important to monitor the patient’s clinical progress during therapy by repeating susceptibility testing to assess for possible acquisition of resistance. Liposomal amphotericin B should be considered as an alternative or additional option if treatment is not effective within five days. Amphotericin B deoxycholate is recommended for neonates and infants under two months of age. For multidrug-resistant *C. auris* isolates, a combination of drugs may be used [28]. However, there are currently insufficient data to determine the most appropriate therapy for pan-resistant strains of *C. auris* [35,104]. These strains have the potential to develop resistance to all three main classes of antifungal agents (echinocandins, amphotericin B, and azoles), even during the course of clinical treatment. Recent studies indicate that combining different antifungal medications, such as azoles with echinocandins, polyenes with 5-flucytosine, or caspofungin with posaconazole, may be effective against pan-resistant strains of *C. auris* [105]. Rezafungin, ibrexafungerp, and fosmanogepix are three compounds currently in late-stage clinical development [106]. Rezafungin is a new echinocandin with a prolonged half-life. Ibrexafungerp is a first-in-class triterpenoid and fosmanogepix is a novel Gwt1 enzyme inhibitor. These are promising candidates for the treatment of invasive candidiasis, including multidrug-resistant isolates of *C. auris* [106].

### Strategies for Preventing the Transmission and Colonisation of C. auris in Healthcare Settings

*Candida auris* can persist and survive for extended periods, particularly in healthcare settings, due to its ability to grow at higher temperatures and tolerate high salt concentrations. These unique characteristics of the fungus allow it to thrive in such environments [10]. Outbreak investigations have shown that *C. auris* isolates from patients can contaminate various surfaces, even those not directly in contact with the patient. These surfaces include ventilators, floors, patient bed trolleys, chairs, and window sills [64]. Therefore, proper decontamination of non-disposable biomedical products and equipment prior to reuse with another patient is essential in order to control fungal transmission.

The importance of implementing strict infection prevention and control measures is demonstrated by the prolonged persistence of *C. auris* on surfaces and its rapid colonisation of patients [30]. A wide variety of products and experimental methods have been tested to disinfect environmental surfaces contaminated with *C. auris* [107]. However, the findings of these studies have not always been consistent [108]. Several studies have demonstrated the effectiveness of Chlor-Clean and Haz-Tab, which are chlorine-based products with concentrations of 1000 ppm and 10,000 ppm, respectively, in eradicating clinical isolates of *C. auris* within 3 min of contact [109]. Further studies have shown that the use of 1% and 2% NaOCl is highly effective in completely eliminating *C. auris* from cellulose surfaces, but not from steel or plastic surfaces, after a 10 min contact time [110]. In addition, most NaOCl-based commercial products were found to be ineffective against *C. auris* biofilms [110]. The effectiveness of removing *C. auris* from various surfaces, including stainless steel, porcelain, plastic, and glass, has been achieved using a combination of peracetic acid and NaOCl [111]. Vaporised hydrogen peroxide is a commonly used method for environmental decontamination. However, there is currently insufficient evidence to support its effectiveness against *C. auris* in healthcare facilities [110]. Therefore, its use is recommended as an additional safety measure in conjunction with other cleaning and disinfection methods. Although quaternary ammonium compounds are frequently used as disinfectants in healthcare settings, their effectiveness against *C. auris* has not yet been proven, and their use is currently not recommended [30]. Infection control precautions should be consistent, as *C. auris* can be transmitted regardless of whether a person is infected or colonised [10]. It is widely recommended to screen those who have been exposed to or have had close contact with confirmed *C. auris* cases. Successful outbreak management was achieved through the active screening of potentially exposed patients, followed by strict infection control measures [112].

## 3. Conclusions

Experimental mouse models have shown that *Candida auris* spreads more easily and can be more resistant than other *Candida* species. It is important to note, however, that resistance does not necessarily indicate greater virulence. *C. auris* is an important example of emerging fungal pathogens becoming increasingly resistant. Factors such as climate change may be contributing to the emergence of this pathogen. The success of the pathogen as a nosocomial infection is attributed to its ability to persist for long periods in ICUs. To tackle this problem, it is important to have more antifungal agents in development, as there are currently only a limited number available. Additionally, diagnostics play a crucial role. A point-of-care test conducted outside of a laboratory setting would have a significant impact. The results would be available quickly, allowing for timely preventive measures to be taken and making screening more efficient. Although it is generally advised that colonisation treatment should not be initiated without clear evidence of infection, it is nonetheless important to ascertain the existence of products capable of reducing the skin burden, thereby preventing infection in the patient and limiting transmission to others.

To prevent infection, healthcare facilities should conduct surveillance by monitoring and identifying patients’ medical records. For example, in the event that a patient tests positive for *C. auris* and is readmitted to the facility, clinicians can be alerted to implement appropriate transmission-related precautions without the necessity for re-screening. In certain healthcare facilities, disease has spread after admitting cases, while others have struggled to contain large epidemics. It is hoped that the knowledge gained about *C. auris* and the improvements made, particularly in infection control, will prevent the spread of future outbreaks.

## Figures and Tables

**Table 1:** Cases of *C. auris* infection EU/EEA *, 2018–2021.

Country	2018	2019	2020	2021
Austria	1	0	2	1
Belgium	0	3	0	1
Denmark	0	0	0	2
Finland	0	0	0	1
France	0	3	4	4
Germany	2	3	5	10
Greek	0	3	13	58
Ireland	0	0	0	1
Italy	0	1	49	242
Malta	0	0	0	0
Norway	1	0	0	2
Spain	230	135	260	331
Sweeden	0	0	1	1
The Netherlands	2	1	1	1

*, EEA: European Economic Area; EU: European Union.

## Data Availability

Not applicable.

## References

[1] Mancuso G., Midiri A., Gerace E., Biondo C. (2022). Role of the innate immune system in host defence against fungal infections. Eur. Rev. Med. Pharmacol. Sci..

[2] Rodrigues M.L., Nosanchuk J.D. (2023). Recognition of fungal priority pathogens: What next?. PLoS Negl. Trop. Dis..

[3] Parums D.V. (2022). Editorial: The World Health Organization (WHO) Fungal Priority Pathogens List in Response to Emerging Fungal Pathogens During the COVID-19 Pandemic. Med. Sci. Monit..

[4] Zhang Z., Bills G.F., An Z. (2023). Advances in the treatment of invasive fungal disease. PLoS Pathog..

[5] WHO (2022). WHO Fungal Priority Pathogens List to Guide Research, Development and Public Health Action. https://www.who.int/publications/i/item/9789240060241.

[6] Fang W., Wu J., Cheng M., Zhu X., Du M., Chen C., Liao W., Zhi K., Pan W. (2023). Diagnosis of invasive fungal infections: Challenges and recent developments. J. Biomed. Sci..

[7] Chowdhary A., Jain K., Chauhan N. (2023). *Candida auris* Genetics and Emergence. Annu. Rev. Microbiol..

[8] Pappas P.G., Lionakis M.S., Arendrup M.C., Ostrosky-Zeichner L., Kullberg B.J. (2018). Invasive candidiasis. Nat. Rev. Dis. Primers.

[9] Abdel-Hamid R.M., El-Mahallawy H.A., Abdelfattah N.E., Wassef M.A. (2023). The impact of increasing non-albicans Candida trends on diagnostics in immunocompromised patients. Braz. J. Microbiol..

[10] Ahmad S., Asadzadeh M. (2023). Strategies to Prevent Transmission of *Candida auris* in Healthcare Settings. Curr. Fungal Infect. Rep..

[11] Colombo A.L., Junior J.N.A., Guinea J. (2017). Emerging multidrug-resistant Candida species. Curr. Opin. Infect. Dis..

[12] Carty J., Chowdhary A., Bernstein D., Thangamani S. (2023). Tools and techniques to identify, study, and control *Candida auris*. PLoS Pathog..

[13] Satoh K., Makimura K., Hasumi Y., Nishiyama Y., Uchida K., Yamaguchi H. (2009). *Candida auris* sp. nov., a novel ascomycetous yeast isolated from the external ear canal of an inpatient in a Japanese hospital. Microbiol. Immunol..

[14] Lee W.G., Shin J.H., Uh Y., Kang M.G., Kim S.H., Park K.H., Jang H.C. (2011). First three reported cases of nosocomial fungemia caused by *Candida auris*. J. Clin. Microbiol..

[15] Crea F., Codda G., Orsi A., Battaglini A., Giacobbe D.R., Delfino E., Ungaro R., Marchese A. (2019). Isolation of *Candida auris* from invasive and non-invasive samples of a patient suffering from vascular disease, Italy, July 2019. Eurosurveillance.

[16] Oh B.J., Shin J.H., Kim M.N., Sung H., Lee K., Joo M.Y., Shin M.G., Suh S.P., Ryang D.W. (2011). Biofilm formation and genotyping of *Candida haemulonii*, *Candida pseudohaemulonii*, and a proposed new species (*Candida auris*) isolates from Korea. Med. Mycol..

[17] Desnos-Ollivier M., Fekkar A., Bretagne S. (2021). Earliest case of *Candida auris* infection imported in 2007 in Europe from India prior to the 2009 description in Japan. J. Mycol. Medicale.

[18] Lamoth F., Kontoyiannis D.P. (2018). The *Candida auris* Alert: Facts and Perspectives. J. Infect. Dis..

[19] Chowdhary A., Sharma C., Duggal S., Agarwal K., Prakash A., Singh P.K., Jain S., Kathuria S., Randhawa H.S., Hagen F. (2013). New clonal strain of *Candida auris*, Delhi, India. Emerg. Infect. Dis..

[20] Forsberg K., Woodworth K., Walters M., Berkow E.L., Jackson B., Chiller T., Vallabhaneni S. (2019). *Candida auris*: The recent emergence of a multidrug-resistant fungal pathogen. Med. Mycol..

[21] Sticchi C., Raso R., Ferrara L., Vecchi E., Ferrero L., Filippi D., Finotto G., Frassinelli E., Silvestre C., Zozzoli S. (2023). Increasing Number of Cases Due to *Candida auris* in North Italy, July 2019–December 2022. J. Clin. Med..

[22] van Schalkwyk E., Mpembe R.S., Thomas J., Shuping L., Ismail H., Lowman W., Karstaedt A.S., Chibabhai V., Wadula J., Avenant T. (2019). Epidemiologic Shift in Candidemia Driven by *Candida auris*, South Africa, 2016-2017(1). Emerg. Infect. Dis..

[23] Chakrabarti A., Sood P., Rudramurthy S.M., Chen S., Kaur H., Capoor M., Chhina D., Rao R., Eshwara V.K., Xess I. (2015). Incidence, characteristics and outcome of ICU-acquired candidemia in India. Intensive Care Med..

[24] Du H., Bing J., Hu T., Ennis C.L., Nobile C.J., Huang G. (2020). *Candida auris*: Epidemiology, biology, antifungal resistance, and virulence. PLoS Pathog..

[25] Sabino R., Verissimo C., Pereira A.A., Antunes F. (2020). *Candida auris*, an Agent of Hospital-Associated Outbreaks: Which Challenging Issues Do We Need to Have in Mind?. Microorganisms.

[26] Sharp A., Muller-Pebody B., Charlett A., Patel B., Gorton R., Lambourne J., Cummins M., Alcolea-Medina A., Wilks M., Smith R. (2021). Screening for *Candida auris* in patients admitted to eight intensive care units in England, 2017 to 2018. Eurosurveillance.

[27] Yadav A., Singh A., Wang Y., Haren M.H.V., Singh A., de Groot T., Meis J.F., Xu J., Chowdhary A. (2021). Colonisation and Transmission Dynamics of *Candida auris* among Chronic Respiratory Diseases Patients Hospitalised in a Chest Hospital, Delhi, India: A Comparative Analysis of Whole Genome Sequencing and Microsatellite Typing. J. Fungi.

[28] Cristina M.L., Spagnolo A.M., Sartini M., Carbone A., Oliva M., Schinca E., Boni S., Pontali E. (2023). An Overview on *Candida auris* in Healthcare Settings. J. Fungi.

[29] Garcia-Bustos V., Cabanero-Navalon M.D., Ruiz-Sauri A., Ruiz-Gaitan A.C., Salavert M., Tormo M.A., Peman J. (2021). What Do We Know about *Candida auris*? State of the Art, Knowledge Gaps, and Future Directions. Microorganisms.

[30] Ahmad S., Alfouzan W. (2021). *Candida auris*: Epidemiology, Diagnosis, Pathogenesis, Antifungal Susceptibility, and Infection Control Measures to Combat the Spread of Infections in Healthcare Facilities. Microorganisms.

[31] Khojasteh S., Jafarzdeh J., Hosseini S.A., Haghani I., Turki H., Aghaei Gharehbolagh S., Abastabar M., Mahmoudi S. (2022). *Candida auris* and COVID-19: A health threatening combination. Curr. Med. Mycol..

[32] Chowdhary A., Sharma A. (2020). The lurking scourge of multidrug resistant *Candida auris* in times of COVID-19 pandemic. J. Glob. Antimicrob. Resist..

[33] Nwachukwu K.C., Nwarunma E., David Uchenna C., Chinyere Ugbogu O. (2023). Enablers of *Candida auris* persistence on medical devices and their mode of eradication. Curr. Med. Mycol..

[34] Logan A., Wolfe A., Williamson J.C. (2022). Antifungal Resistance and the Role of New Therapeutic Agents. Curr. Infect. Dis. Rep..

[35] Ademe M., Girma F. (2020). *Candida auris*: From Multidrug Resistance to Pan-Resistant Strains. Infect. Drug Resist..

[36] Ong C.W., Chen S.C., Clark J.E., Halliday C.L., Kidd S.E., Marriott D.J., Marshall C.L., Morris A.J., Morrissey C.O., Roy R. (2019). Diagnosis, management and prevention of *Candida auris* in hospitals: Position statement of the Australasian Society for Infectious Diseases. Intern. Med. J..

[37] Spivak E.S., Hanson K.E. (2018). *Candida auris*: An Emerging Fungal Pathogen. J. Clin. Microbiol..

[38] Lone S.A., Ahmad A. (2019). *Candida auris*—The growing menace to global health. Mycoses.

[39] Tharp B., Zheng R., Bryak G., Litvintseva A.P., Hayden M.K., Chowdhary A., Thangamani S. (2023). Role of Microbiota in the Skin Colonization of *Candida auris*. mSphere.

[40] Cortegiani A., Misseri G., Fasciana T., Giammanco A., Giarratano A., Chowdhary A. (2018). Epidemiology, clinical characteristics, resistance, and treatment of infections by *Candida auris*. J. Intensive Care.

[41] Osei Sekyere J. (2018). *Candida auris*: A systematic review and meta-analysis of current updates on an emerging multidrug-resistant pathogen. MicrobiologyOpen.

[42] Ashkenazi-Hoffnung L., Rosenberg Danziger C. (2023). Navigating the New Reality: A Review of the Epidemiological, Clinical, and Microbiological Characteristics of *Candida auris*, with a Focus on Children. J. Fungi.

[43] Caceres D.H., Forsberg K., Welsh R.M., Sexton D.J., Lockhart S.R., Jackson B.R., Chiller T. (2019). *Candida auris*: A Review of Recommendations for Detection and Control in Healthcare Settings. J. Fungi.

[44] Al-Dorzi H.M., Sakkijha H., Khan R., Aldabbagh T., Toledo A., Ntinika P., Al Johani S.M., Arabi Y.M. (2020). Invasive Candidiasis in Critically Ill Patients: A Prospective Cohort Study in Two Tertiary Care Centers. J. Intensive Care Med..

[45] Lesan A., Man M.A., Nemes R.M., Harsovescu T., Tudorache I.S., Boca-Mahler B., Pop C.M. (2019). Serum Interleukin 4 and 6 Levels Measured Using the ELISA Method in Patients with Acquired Bronchiectasis Compared to Healthy Subjects. An Anti-Inflammatory and pro-Inflammatory Relation. Rev. Chim..

[46] Jackson B.R., Chow N., Forsberg K., Litvintseva A.P., Lockhart S.R., Welsh R., Vallabhaneni S., Chiller T. (2019). On the Origins of a Species: What Might Explain the Rise of *Candida auris*?. J. Fungi.

[47] Rapti V., Iliopoulou K., Poulakou G. (2023). The Gordian Knot of C. auris: If You Cannot Cut It, Prevent It. Pathogens.

[48] Alfaifi A., Brooks J.K., Jabra-Rizk M.A., Meiller T.F., Sultan A.S. (2023). Does *Candida auris* colonize the oral cavity? A retrospective institutional experience. Oral Dis..

[49] Bing J., Wang S., Xu H., Fan S., Du H., Nobile C.J., Huang G. (2022). A case of *Candida auris* candidemia in Xiamen, China, and a comparative analysis of clinical isolates in China. Mycology.

[50] Chybowska A.D., Childers D.S., Farrer R.A. (2020). Nine Things Genomics Can Tell Us About *Candida auris*. Front. Genet..

[51] Sharma C., Kadosh D. (2023). Perspective on the origin, resistance, and spread of the emerging human fungal pathogen *Candida auris*. PLoS Pathog..

[52] Taghizadeh Armaki M., Mahdavi Omran S., Kiakojuri K., Khojasteh S., Jafarzadeh J., Tavakoli M., Badali H., Haghani I., Shokohi T., Taghi Hedayati M. (2021). First fluconazole-resistant *Candida auris* isolated from fungal otitis in Iran. Curr. Med. Mycol..

[53] Jeffery-Smith A., Taori S.K., Schelenz S., Jeffery K., Johnson E.M., Borman A., Candida auris Incident Management T., Manuel R., Brown C.S. (2018). *Candida auris*: A Review of the Literature. Clin. Microbiol. Rev..

[54] Hu S., Zhu F., Jiang W., Wang Y., Quan Y., Zhang G., Gu F., Yang Y. (2021). Retrospective Analysis of the Clinical Characteristics of *Candida auris* Infection Worldwide from 2009 to 2020. Front. Microbiol..

[55] Al-Hatmi A.M.S., Mohsin J., Al-Huraizi A., Khamis F. (2021). COVID-19 associated invasive candidiasis. J. Infect..

[56] Tsai C.S., Lee S.S., Chen W.C., Tseng C.H., Lee N.Y., Chen P.L., Li M.C., Syue L.S., Lo C.L., Ko W.C. (2023). COVID-19-associated candidiasis and the emerging concern of *Candida auris* infections. J. Microbiol. Immunol. Infect..

[57] Thomsen J., Abdulrazzaq N.M., Oulhaj A., Nyasulu P.S., Alatoom A., Denning D.W., Al Dhaheri F., Consortium U.A.S., Menezes G.A., Moubareck C.A. (2023). Emergence of highly resistant *Candida auris* in the United Arab Emirates: A retrospective analysis of evolving national trends. Front. Public Health.

[58] Senok A., Alfaresi M., Khansaheb H., Nassar R., Hachim M., Al Suwaidi H., Almansoori M., Alqaydi F., Afaneh Z., Mohamed A. (2021). Coinfections in Patients Hospitalized with COVID-19: A Descriptive Study from the United Arab Emirates. Infect. Drug Resist..

[59] Bagheri Lankarani K., Akbari M., Tabrizi R., Vali M., Sekhavati E., Heydari S.T., Khodadadi H., Ahmadizar F. (2022). *Candida auris*: Outbreak fungal pathogen in COVID-19 pandemic: A systematic review and meta-analysis. Iran. J. Microbiol..

[60] Magnasco L., Mikulska M., Giacobbe D.R., Taramasso L., Vena A., Dentone C., Dettori S., Tutino S., Labate L., Di Pilato V. (2021). Spread of Carbapenem-Resistant Gram-Negatives and *Candida auris* during the COVID-19 Pandemic in Critically Ill Patients: One Step Back in Antimicrobial Stewardship?. Microorganisms.

[61] Mulet Bayona J.V., Tormo Palop N., Salvador Garcia C., Fuster Escriva B., Chanza Avino M., Ortega Garcia P., Gimeno Cardona C. (2021). Impact of the SARS-CoV-2 Pandemic in Candidaemia, Invasive Aspergillosis and Antifungal Consumption in a Tertiary Hospital. J. Fungi.

[62] Pfaller M.A., Diekema D.J., Turnidge J.D., Castanheira M., Jones R.N. (2019). Twenty Years of the SENTRY Antifungal Surveillance Program: Results for Candida Species from 1997–2016. Open Forum Infect. Dis..

[63] Lockhart S.R., Etienne K.A., Vallabhaneni S., Farooqi J., Chowdhary A., Govender N.P., Colombo A.L., Calvo B., Cuomo C.A., Desjardins C.A. (2017). Simultaneous Emergence of Multidrug-Resistant *Candida auris* on 3 Continents Confirmed by Whole-Genome Sequencing and Epidemiological Analyses. Clin. Infect. Dis..

[64] Chakrabarti A., Sood P. (2021). On the emergence, spread and resistance of *Candida auris*: Host, pathogen and environmental tipping points. J. Med. Microbiol..

[65] Chow N.A., Munoz J.F., Gade L., Berkow E.L., Li X., Welsh R.M., Forsberg K., Lockhart S.R., Adam R., Alanio A. (2020). Tracing the Evolutionary History and Global Expansion of *Candida auris* Using Population Genomic Analyses. mBio.

[66] Rossato L., Colombo A.L. (2018). *Candida auris*: What Have We Learned About Its Mechanisms of Pathogenicity?. Front. Microbiol..

[67] Kim J.S., Lee K.T., Lee M.H., Cheong E., Bahn Y.S. (2021). Adenylyl Cyclase and Protein Kinase A Play Redundant and Distinct Roles in Growth, Differentiation, Antifungal Drug Resistance, and Pathogenicity of *Candida auris*. mBio.

[68] Summary W. (2014). The Influence of Global Environmental Change on Infectious Disease Dynamics: Workshop Summary.

[69] Casadevall A., Kontoyiannis D.P., Robert V. (2021). Environmental *Candida auris* and the Global Warming Emergence Hypothesis. mBio.

[70] Garcia-Bustos V. (2024). Is *Candida auris* the first multidrug-resistant fungal zoonosis emerging from climate change?. mBio.

[71] White T.C., Esquivel B.D., Rouse Salcido E.M., Schweiker A.M., Dos Santos A.R., Gade L., Petro E., KuKanich B., KuKanich K.S. (2024). *Candida auris* detected in the oral cavity of a dog in Kansas. mBio.

[72] Iguchi S., Itakura Y., Yoshida A., Kamada K., Mizushima R., Arai Y., Uzawa Y., Kikuchi K. (2019). *Candida auris*: A pathogen difficult to identify, treat, and eradicate and its characteristics in Japanese strains. J. Infect. Chemother..

[73] Thatchanamoorthy N., Rukumani Devi V., Chandramathi S., Tay S.T. (2022). *Candida auris*: A Mini Review on Epidemiology in Healthcare Facilities in Asia. J. Fungi.

[74] Berrio I., Caceres D.H., Coronell R.W., Salcedo S., Mora L., Marin A., Varon C., Lockhart S.R., Escandon P., Berkow E.L. (2021). Bloodstream Infections with *Candida auris* among Children in Colombia: Clinical Characteristics and Outcomes of 34 Cases. J. Pediatr. Infect. Dis. Soc..

[75] Zamith-Miranda D., Heyman H.M., Couvillion S.P., Cordero R.J.B., Rodrigues M.L., Nimrichter L., Casadevall A., Amatuzzi R.F., Alves L.R., Nakayasu E.S. (2021). Comparative Molecular and Immunoregulatory Analysis of Extracellular Vesicles from *Candida albicans* and *Candida auris*. mSystems.

[76] Horton M.V., Johnson C.J., Zarnowski R., Andes B.D., Schoen T.J., Kernien J.F., Lowman D., Kruppa M.D., Ma Z., Williams D.L. (2021). *Candida auris* Cell Wall Mannosylation Contributes to Neutrophil Evasion through Pathways Divergent from *Candida albicans* and *Candida glabrata*. mSphere.

[77] Bergeron G., Bloch D., Murray K., Kratz M., Parton H., Ackelsberg J., Antwi M., Del Rosso P., Dorsinville M., Kubinson H. (2021). *Candida auris* Colonization After Discharge to a Community Setting: New York City, 2017–2019. Open Forum Infect. Dis..

[78] Schwartz I.S., Smith S.W., Dingle T.C. (2018). Something wicked this way comes: What health care providers need to know about *Candida auris*. Can. Commun. Dis. Rep. = Relev. Des Mal. Transm. Au Can..

[79] Tian S., Bing J., Chu Y., Chen J., Cheng S., Wang Q., Zhang J., Ma X., Zhou B., Liu L. (2021). Genomic epidemiology of *Candida auris* in a general hospital in Shenyang, China: A three-year surveillance study. Emerg. Microbes Infect..

[80] Gonzalez-Duran E., Contreras-Perez C.U., Caceres D.H., Rios-Rosas C., Pinon-Ortega J.J., Tellez-Saucedo M.D., Marin-Suro E.S., Wong-Arambula C.E., Moreno-Escobar E.A., Ramirez-Gonzalez J.E. (2022). The use of readily available laboratory tests for the identification of the emerging yeast *Candida auris* in Mexico. Arch. Microbiol..

[81] Fasciana T., Cortegiani A., Ippolito M., Giarratano A., Di Quattro O., Lipari D., Graceffa D., Giammanco A. (2020). *Candida auris*: An Overview of How to Screen, Detect, Test and Control This Emerging Pathogen. Antibiotics.

[82] Marathe A., Zhu Y., Chaturvedi V., Chaturvedi S. (2022). Utility of CHROMagar Candida Plus for presumptive identification of *Candida auris* from surveillance samples. Mycopathologia.

[83] Carolus H., Jacobs S., Lobo Romero C., Deparis Q., Cuomo C.A., Meis J.F., Van Dijck P. (2021). Diagnostic Allele-Specific PCR for the Identification of *Candida auris* Clades. J. Fungi.

[84] Bumbrah G.S., Jain S., Singh S., Fatima Z., Hameed S. (2023). Diagnostic Efficacy of LAMP Assay for Human Fungal Pathogens: A Systematic Review and Meta-analysis. Curr. Fungal Infect. Rep..

[85] Geremia N., Brugnaro P., Solinas M., Scarparo C., Panese S. (2023). *Candida auris* as an Emergent Public Health Problem: A Current Update on European Outbreaks and Cases. Healthcare.

[86] Narayanan A., Selvakumar P., Siddharthan R., Sanyal K. (2022). ClaID: A Rapid Method of Clade-Level Identification of the Multidrug Resistant Human Fungal Pathogen *Candida auris*. Microbiol. Spectr..

[87] Katsiari M., Mavroidi A., Kesesidis N., Palla E., Zourla K., Ntorlis K., Konstantinidis K., Laskou M., Strigklis K., Sakkalis A. (2023). Emergence of Clonally-Related South Asian Clade I Clinical Isolates of *Candida auris* in a Greek COVID-19 Intensive Care Unit. J. Fungi.

[88] Chaabane F., Graf A., Jequier L., Coste A.T. (2019). Review on Antifungal Resistance Mechanisms in the Emerging Pathogen *Candida auris*. Front. Microbiol..

[89] Shaban S., Patel M., Ahmad A. (2020). Improved efficacy of antifungal drugs in combination with monoterpene phenols against *Candida auris*. Sci. Rep..

[90] Czajka K.M., Venkataraman K., Brabant-Kirwan D., Santi S.A., Verschoor C., Appanna V.D., Singh R., Saunders D.P., Tharmalingam S. (2023). Molecular Mechanisms Associated with Antifungal Resistance in Pathogenic Candida Species. Cells.

[91] Fan S., Yue H., Zheng Q., Bing J., Tian S., Chen J., Ennis C.L., Nobile C.J., Huang G., Du H. (2021). Filamentous growth is a general feature of *Candida auris* clinical isolates. Med. Mycol..

[92] Martini C., Torelli R., de Groot T., De Carolis E., Morandotti G.A., De Angelis G., Posteraro B., Meis J.F., Sanguinetti M. (2020). Prevalence and Clonal Distribution of Azole-Resistant Candida parapsilosis Isolates Causing Bloodstream Infections in a Large Italian Hospital. Front. Cell. Infect. Microbiol..

[93] Hosseini S.S., Yadegari M.H., Rajabibazl M., Ghaemi E.A. (2016). Inhibitory effects of carvacrol on the expression of secreted aspartyl proteinases 1-3 in fluconazole-resistant *Candida albicans* isolates. Iran. J. Microbiol..

[94] Banerjee A., Pata J., Sharma S., Monk B.C., Falson P., Prasad R. (2021). Directed Mutational Strategies Reveal Drug Binding and Transport by the MDR Transporters of *Candida albicans*. J. Fungi.

[95] Ahmady L., Gothwal M., Mukkoli M.M., Bari V.K. (2024). Antifungal drug resistance in Candida: A special emphasis on amphotericin B. APMIS: Acta Pathol. Microbiol. Immunol. Scand..

[96] Morace G., Perdoni F., Borghi E. (2014). Antifungal drug resistance in Candida species. J. Glob. Antimicrob. Resist..

[97] Bravo Ruiz G., Lorenz A. (2021). What do we know about the biology of the emerging fungal pathogen of humans *Candida auris*?. Microbiol. Res..

[98] Arendrup M.C., Perlin D.S. (2014). Echinocandin resistance: An emerging clinical problem?. Curr. Opin. Infect. Dis..

[99] Silva L.N., Ramos L.S., Oliveira S.S.C., Magalhaes L.B., Cypriano J., Abreu F., Macedo A.J., Branquinha M.H., Santos A.L.S. (2023). Development of Echinocandin Resistance in *Candida haemulonii*: An Emergent, Widespread, and Opportunistic Fungal Pathogen. J. Fungi.

[100] de Oliveira Santos G.C., Vasconcelos C.C., Lopes A.J.O., de Sousa Cartagenes M.D.S., Filho A., do Nascimento F.R.F., Ramos R.M., Pires E., de Andrade M.S., Rocha F.M.G. (2018). Candida Infections and Therapeutic Strategies: Mechanisms of Action for Traditional and Alternative Agents. Front. Microbiol..

[101] Weerasinghe H., Simm C., Djajawi T.M., Tedja I., Lo T.L., Simpson D.S., Shasha D., Mizrahi N., Olivier F.A.B., Speir M. (2023). *Candida auris* uses metabolic strategies to escape and kill macrophages while avoiding robust activation of the NLRP3 inflammasome response. Cell Rep..

[102] Moreno-Garcia E., Puerta-Alcalde P., Gariup G., Fernandez-Ruiz M., Lopez Cortes L.E., Cuervo G., Salavert M., Merino P., Machado M., Guinea J. (2021). Early Stepdown from Echinocandin to Fluconazole Treatment in Candidemia: A Post Hoc Analysis of Three Cohort Studies. Open Forum Infect. Dis..

[103] Hirayama T., Miyazaki T., Sumiyoshi M., Ito Y., Ashizawa N., Takeda K., Iwanaga N., Takazono T., Yamamoto K., Izumikawa K. (2023). Echinocandin Resistance in *Candida auris* Occurs in the Murine Gastrointestinal Tract Due to FKS1 Mutations. Antimicrob. Agents Chemother..

[104] Singh S., Barbarino A., Youssef E.G., Coleman D., Gebremariam T., Ibrahim A.S. (2023). Protective Efficacy of Anti-Hyr1p Monoclonal Antibody against Systemic Candidiasis Due to Multi-Drug-Resistant *Candida auris*. J. Fungi.

[105] Jacobs S.E., Jacobs J.L., Dennis E.K., Taimur S., Rana M., Patel D., Gitman M., Patel G., Schaefer S., Iyer K. (2022). *Candida auris* Pan-Drug-Resistant to Four Classes of Antifungal Agents. Antimicrob. Agents Chemother..

[106] Lamoth F. (2023). Novel Therapeutic Approaches to Invasive Candidiasis: Considerations for the Clinician. Infect. Drug Resist..

[107] Cadnum J.L., Shaikh A.A., Piedrahita C.T., Jencson A.L., Larkin E.L., Ghannoum M.A., Donskey C.J. (2018). Relative Resistance of the Emerging Fungal Pathogen *Candida auris* and Other Candida Species to Killing by Ultraviolet Light. Infect. Control Hosp. Epidemiol..

[108] Omardien S., Teska P. (2024). Skin and hard surface disinfection against *Candida auris*—What we know today. Front. Med..

[109] Abdolrasouli A., Armstrong-James D., Ryan L., Schelenz S. (2017). In vitro efficacy of disinfectants utilised for skin decolonisation and environmental decontamination during a hospital outbreak with *Candida auris*. Mycoses.

[110] Ku T.S.N., Walraven C.J., Lee S.A. (2018). *Candida auris*: Disinfectants and Implications for Infection Control. Front. Microbiol..

[111] Kean R., Sherry L., Townsend E., McKloud E., Short B., Akinbobola A., Mackay W.G., Williams C., Jones B.L., Ramage G. (2018). Surface disinfection challenges for *Candida auris*: An in-vitro study. J. Hosp. Infect..

[112] Alshamrani M.M., El-Saed A., Mohammed A., Alghoribi M.F., Al Johani S.M., Cabanalan H., Balkhy H.H. (2021). Management of *Candida auris* outbreak in a tertiary-care setting in Saudi Arabia. Infect. Control Hosp. Epidemiol..

